# ReCAN – Dataset for reverse engineering of Controller Area Networks

**DOI:** 10.1016/j.dib.2020.105149

**Published:** 2020-01-22

**Authors:** Mattia Zago, Stefano Longari, Andrea Tricarico, Michele Carminati, Manuel Gil Pérez, Gregorio Martínez Pérez, Stefano Zanero

**Affiliations:** a Dipartimento di Elettronica, Informazione e Bioingegneria, Politecnico di Milano, Milan, Italy; b Department of Information Engineering and Communications, University of Murcia, Murcia, Spain

**Keywords:** Automotive, Controller area network (CAN), Reverse engineering, Dataset

## Abstract

This article details the methodology and the approach used to extract and decode the data obtained from the Controller Area Network (CAN) buses in two personal vehicles and three commercial trucks for a total of 36 million data frames. The dataset is composed of two complementary parts, namely the raw data and the decoded ones. Along with the description of the data, this article also reports both hardware and software requirements to first extract the data from the vehicles and secondly decode the binary data frames to obtain the actual sensors’ data. Finally, to enable analysis reproducibility and future researches, the code snippets that have been described in pseudo-code will be publicly available in a code repository. Motivated enough actors may intercept, interact, and recognize the vehicle data with consumer-grade technology, ultimately refuting, once-again, the security-through-obscurity paradigm used by the automotive manufacturer as a primary defensive countermeasure.

**Table:** Specification Table

Subject area	Engineering, Computer Science
Specific area	Automotive Engineering, Artificial Intelligence
Type of data	CSV files
How data were acquired	Controller Area Network (CAN) buses have been accessed using a standard CAN connector and a CANtact board. The CAN Utils library, publicly available in the Linux Kernel, has been used to intercept the network traffic of the vehicle. Sensors data have been decoded using the state-of-the-art algorithm. The source code for each step of the analysis is publicly available in the repository, as specified below.
Data format	Raw and Filtered
Parameters for data collection	**Cars**: 500k baud rate, connected o the OBD-II port of each vehicle.
	**Trucks**: 500k baud rate, connected both to the OBD-II port and to a second wire into a second CAN bus.
Description of data collection	**Phase 1**: Using consumer-grade hardware, we accessed the Controller Area Network (CAN) buses of five vehicles. CSV files contain the binary sequence for each CANline and identifier in the experiment time window.
	**Phase 2**: Raw data have been decoded and interpreted with well-known and previously validated algorithms to identify the sensors' variables. Decoded CSV files contain the sequence of values for each variable, identifier, and CANline in the experiment time window.
Data source location	Dipartimento di Elettronica, Informazione e Bioingengeria, Politecnico di Milano, Milan, Italy
Data accessibility	Data repository: ReCAN Data - Reverse engineering of Controller Area Networks [1]
	Data identification number: 10.17632/76knkx3fzv
	Direct URL to data: https://data.mendeley.com/datasets/76knkx3fzv
	Source code repository: ReCAN Source - Reverse engineering of Controller Area Networks [2]
	Source code URL: https://github.com/Cyberdefence-Lab-Murcia/ReCAN

**Table:** 

**Value of the Data** • These data endeavor to fulfill the lack of large, continuous, and machine-learning-ready datasets for automotive analysis. • The primary recipient for the data are the academic scientists that focus on machine-learning-driven researches. They might greatly benefit from these freshly generated and carefully reviewed data. • The main usage of this data is twofold: i) the raw data can be used to train unsupervised automatic decoders while ii) the decoded data can be used to power self-optimized intrusion detection systems. • The Controller Area Network (CAN) streams are also decoded and interpreted, such preprocess might provide the scientific community with additional and improved data characterization.

## 1 Data description

**Fig. 1:**
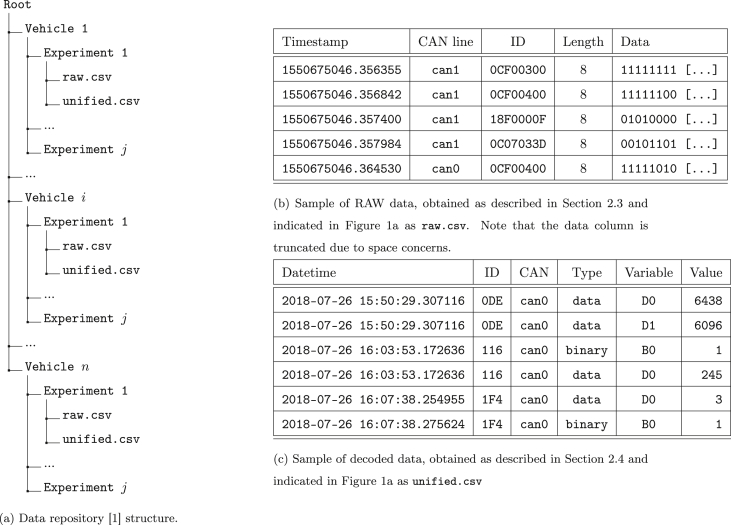
Data repository structure and data samples. (b) Sample of RAW data, obtained as described in Section 2.3 and indicated in Fig. 1a as raw.csv. Note that the data column is truncated due to space concerns. (c) Sample of decoded data, obtained as described in Section 2.4 and indicated in Fig. 1a as unified.csv

**Fig. 2:**
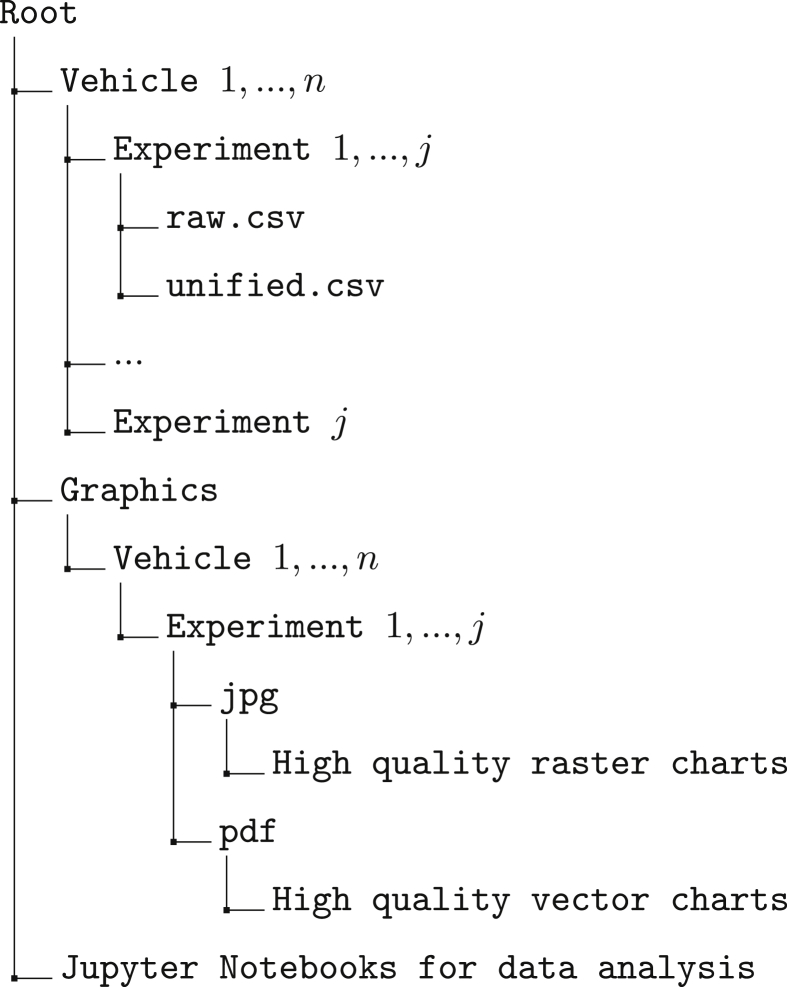
Code repository [2].

This dataset aims to provide two types of data to the scientific community, namely, i) a curated dataset of automotive raw data frames collected from multiple vehicles (raw.csv files in Fig. 1), and, ii) the same data interpreted and decoded (unified.csv files in Fig. 1). Both aspects are necessary to provide a common ground for any Machine Learning (ML) analyzer. The data will be available on Mendeley Data [1]. Moreover, the source code used to extract, decode, and analyze the data is available in a public repository [2]. Figs. 1a and 2 respectively present the structure and contents of the data repository [1] and the code repository [2] and will be described in details in Section 1.1 and Section 1.2 respectively.

### 1.1 Data repository

The data repository is composed of a folder for each experiment, as will be described in Section 2 and summarised in Table 2. For each vehicle, both the raw and the decoded data are stored as CSV files. Fig. 1a presents its structure.

**Table 1:** Dataset composition according to vehicle type.

ID	Vehicle	Type	Connector	FMS
C-1	Alfa Romeo Giulia Veloce	Car	OBD-II	No
C-2	Opel Corsa	Car	OBD-II	No
T-1	Mitsubishi Fuso Canter	Commercial Truck	OBD-II	Yes
T-2	ISUZU M55	Commercial Truck	OBD-II, direct wire access	No
T-3	Piaggio Porter Maxi	Commercial Truck	OBD-II	No

**Table 2:** List of experiments per vehicle.

Vehicle	Test	Experiment time			IDs	Frames	Description
		Date	Start	End			
C-1	#1	2018-07-26	15:15:58	15:35:20	77	3,062,691	city and highway driving
C-1	#2	2018-07-26	15:46:13	15:48:32	76	364,863	city and highway driving
C-1	#3	2018-07-26	15:49:10	15:49:23	76	33,005	repeated brake tests
C-1	#4	2018-07-26	15:50:29	16:10:54	83	3,227,315	city and highway driving
C-1	#5	2018-07-26	16:10:57	16:20:16	83	1,473,625	city and highway driving
C-1	#6	2018-07-26	16:20:20	16:30:59	83	1,684,769	city and highway driving
C-1	#7	2018-07-26	16:53:17	17:10:31	83	2,723,484	city and highway driving
C-1	#8	2019-02-01	16:31:01	16:40:58	82	1,569,776	city and highway driving
C-1	#9	2019-02-01	15:18:55	16:30:36	88	10,942,747	city and highway driving
C-2	#1	2019-10-02	08:54:16	09:22:40	78	3,467,855	city and highway driving
T-1	#1	2019-02-20	16:04:06	16:35:04	31, 47^a^	1,798,602^a^	city and highway driving
T-2	#1	2019-11-08	14:51:57	15:07:43	22	498,721	city driving
T-2	#2	2019-11-08	14:34:33	14:43:20	22	263,269	vehicle not moving
T-3	#1	2019-11-08	11:48:56	12:14:58	23	1,729,623	city driving
T-3	#2	2019-11-08	11:16:55	11:23:42	19	2,795,321	vehicle not moving test 1
T-3	#3	2019-11-08	12:57:48	13:42:52	23	2,795,321	vehicle not moving test 2

a For this experiment, there are included both can0 and can1 lines.

The first set of data, namely the raw dataset, consists of a list of data frames’ data field augmented with the timestamp (POSIX time), the CAN line identifier, and the Engine Control Unit (ECU) identifier (hexadecimal value). An excerpt of data is available in Fig. 1b (the binary sequences are truncated to improve their readability).

The second set of data, namely the unified dataset, consists of the list of all variables with their values for each CANline and ECU identifier. In this case, instead of having the timestamps as POSIX time, the values are expressed in a human-readable format (yyyy-MM-dd HH:mm:ss.S). Fig. 1c provides an excerpt of these unified data.

Since the analysis is heuristic-based and limited to a specific time window, it is possible that some ECU identifiers exist in the raw data, but not in the decoded version. This situation happens whenever there is a combination of CANline and ECU identifiers that present constant values across all recorded data frames. For example, by looking at the Opel Corsa data, it appears that the identifiers 0D1, 0F1, 139, 148, 17D, 182 (among others) are providing data frames that have constant values, thus they have been ignored by the heuristic described in Section 2.

Table 1 presents the vehicles included in the dataset, reporting the vehicle identifier, the type, the connector used and whether there was the Fleet Management Systems Interface (FMS) available. Similarly, Table 2 reports for each vehicle the experiments that were performed with their time windows and description. The table is also providing a summary regarding the number of ECU identifiers and data frames.

### 1.2 Code repository

The code repository is composed of three main parts, as depicted in Fig. 2. Firstly, a compressed copy of the data is available in the data folder, effectively mirroring the contents of the data repository [1]. Secondly, in the repository root, there is the Jupyter Notebook (python scripts) used to extract, decode, and analyze the data. Lastly, the graphics folder contains for each vehicle all the pictures generated by the analysis that have been used by the human expert to provide feedback on the process. In Section 2, we show examples of such charts and figures to support the dataset description.

Finally, Figures from 3 to 9 and Table 3 have been used as support to justify the methodology and the experiments assumptions.

**Table 3:**
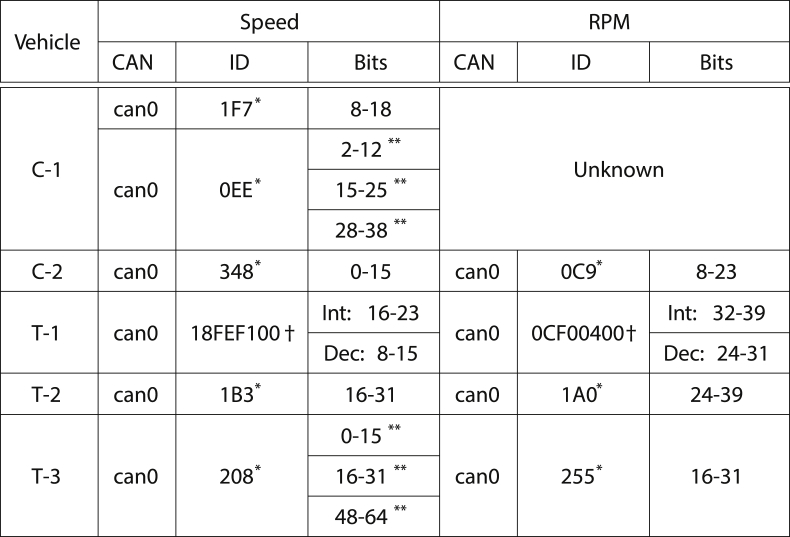
Main identifiers for each vehicle.

^†^ Obtained by FMS.

^∗^ Manually identified.

^∗∗^ These variables appear to be replicated multiple times in the data frame.

## 2 Experimental design, materials, and methods

The vehicle data can be accessed through a connection with the physical CAN network, which nowadays is mainly provided by a standard SAE J1962 [3] On-Board Diagnostics (OBD) connector [4]. CAN communication is based on four different kinds of frames, Namely:

• **Data frames** carry data from a transmitting ECU. For example, a frame containing the steering wheel angle.
• **Remote frames** are used to request the transmission of a data frame, using the ID to signal which frame is needed. For example, A is the unit responsible for transmitting messages with ID 0x01, another unit B can send a remote frame with ID 0x01 to request A to send a data frame. Usually, these frames are not used, as data frames are typically sent at specific time intervals.
• **Error frames** are transmitted when bus errors occur, *e.g.*, when badly formed frames are transmitted.
• **Overload frames** signal a delay of the next data frame because the transmitting ECU is overloaded at the moment.

In the logs, the visible CAN traffic is composed by *data* and *remote* frames (which are not often used in standard CAN communication). In our case, remote frames were rarely detected and have been dropped in the final version of the data. *Error* and *overload* frames serve only as control infrastructure and are not usually relayed from the CAN controller to higher-layer applications such as the CAN-to-USB interface drivers. Since they do not carry information, they have been excluded from this analysis.

**Fig. 3:**
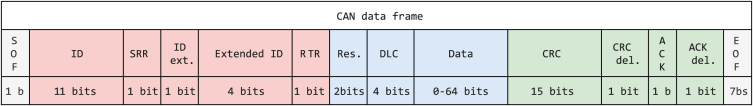
CAN data frame.

Data frames have a standard and well-defined packet structure, as indicated by the ISO 15765-2 standard [5]. Fig. 3 presents a schematic view of the frame bytes where it is possible to notice both the ID and the data fields. The internal structure and encoding of the data fields are proprietary, and a decoding manual is generally not available to the public. Nevertheless, in the case of commercial vehicles, like some of the trucks identified in the following paragraphs, the FMS cheat sheet has been made available by a third party company that provides these services (as specified in the SAE J1939 standard [6]).

### 2.1 Experiments and data fields

The experimental evaluation has been conducted on five different vehicles, and more than 38 million frames have been collected to provide coverage and generality. Table 1 reports the complete list of vehicles with their type and the connector used to access the CAN lines. As shown in Table 1, there are both consumer cars and commercial trucks. Table 2 reports the list of the experiments with their time windows and the number of data frames collected.

Although there are no specific differences between the two categories (they both operate within the (OBD)-II standard, [3], Chapter 2.2 *Related Publications*]), they do differ in terms of driveability and general drivers’ behavior. Nevertheless, our analysis shows that standard sensors, such as speed and Revolutions per minute (RPM), have analog series properties.

Unfortunately, the conditions, the timing and the configuration of each vehicle differs from the others. However, as presented in Table 3 it was possible to identify some most important signals sent by the vehicles, *i*.*e*., speed, RPM and wheels position.

Experiment EX-1-B with the vehicle Alfa Giulia (C-1) consists in a series of sudden and abrupt hard braking with the purpose of activating the safety devices on the vehicle (such as the anti-lock braking system (ABS) and the traction control system (TCS)).

### 2.2 Framework architecture

**Fig. 4:**
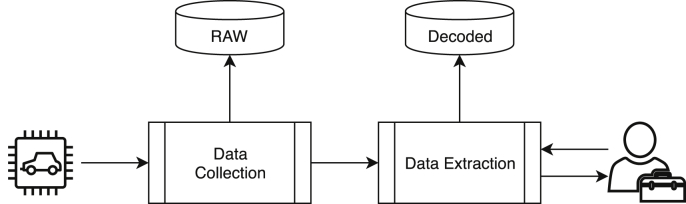
Architecture of the collection framework including both the vehicle and the direct human intervention.

In Fig. 4 the architecture of the framework is illustrated. The process starts with a vehicle's test drive, as described in Table 2. As described in detail in Section 2.3, the Data Collection module consists in both hardware and software elements that act as a probe for extracting CAN frames. This data is stored and published as raw data (see raw.csv in Fig. 1a).

The raw data is then preprocessed in order to calculate statistical features to help decode the frames by the Data Extraction module, as described in Section 2.4. The human expert can provide insights and feedback regarding the decoding process, for example, by identifying errors or introducing more sophisticated heuristic to distinguish individual cases.

### 2.3 Data collection

The process of data collection consists of connecting a laptop to the CAN network(s) in order to intercept the traffic during the experiments. In the following paragraphs, both the hardware and software requirements will be listed. As mentioned above, the scope of the collection phase is to obtain the content of the data frames, obtaining a raw data file for each vehicle.

#### 2.3.1 Hardware requirements

CANs are generally accessible through the OBD-II connector (either type A or type B depending on the manufacturer of the vehicle). However, as also presented in Table 1, some CAN lines may not be directly accessible from the connector. Thus a physical toolkit to connect to the wires is required. To be precise, not all OBD-II connectors available for the consumers market provide raw access to the CAN lines. The totality of wireless devices and most of the wired devices that offer a OBD-II connector are ELM327 microcontrollers that request diagnostic messages and translate the vehicle response, not providing any CAN-to-USB interface.

An open-source hardware device specifically made to access the CAN-to-USB interface is represented by the CANtact device [7].

#### 2.3.2 Software requirements

In terms of software requirements, the Linux kernel already provides the libraries CAN Utils [8], which suffice to interact with the devices and the vehicle's CAN network. To be more precise, in order to retrieve the CAN traffic, it is necessary to bring up the network interface that provides connectivity. This can be done through the command slcand [options] <tty> [CAN interface], with some tweaks regarding the CAN bitrate option values. Having a successful connection with the vehicle, the CAN traffic can be retrieved with the command candump [options] <CAN interface>. We used the option -t a to force the POSIX timestamp of the data to be absolute.

For example, the output of the candump command looks like:

(1573208215.472159) can0 300 [8] 64 00 00 00 00 00 00 00.

However, the raw CSV files have a slightly different format. The conversion is done y converting each byte of the frame's data to binary and padding it with zeros to reach exactly 8 bits length.

### 2.4 Data extraction

Despite the lack of signal-semantic knowledge, as reported in Section 2.3, the data have been analyzed to extrapolate characteristics that eventually led to a decoded interpretation of the sensors data. As will be described in this section, by analyzing the data through multiple prisms, it was possible to pinpoint common structures and, in general, the behavior of the data content. That is to say, the data field (see Fig. 3) has often a substructure of its own, often following a proprietary format. For example, a single data frame may contain both the vehicle speed and the engine revolutions per minute as 32-bit integers.

As suggested by Markovitz [9] and Marchetti [10], among others, it is possible to guess the internal structure of the data field by looking at the variability of the bits. Specifically, a sequence of one or more bits always at zero may indicate a field separator.

Leveraging the knowledge of the identifiers, each vehicle trace is separated in sub-traces, one for each ID and CANline, *i*.*e*., each subtrace includes all (and only) the frames for a specific pair ID-CANline, in the same order as they arrived. For each sub-trace, the first operation that serves as the backbone for the analysis is the extraction of the bitflip value for each bit in the sequence. Following [10], we define the bitflip function as the ratio between the number of bit's value changes and the number of received packages so far. That is to say, let us consider the ith bit of the nth packet, then the bitflip is defined as the number of flips of ith bit divided by n. Within the scope of this research, the bitflips are calculated only over the whole sequence of data frames. As described in Alg. 1, the algorithm provides both the sequence of bitflips for any given (sorted) list of data frames and their proportional value. As an example, the values range from 0 for constant bits (*i*.*e*., there are no changes) to 1 for those bits that constantly changes the value (*i*.*e*., each bit is different from the one in the previous data frame). Moreover, following [10] we define for any bitflip *b* the magnitude mag(b) as described in Equation (1):

(1) $$\text{mag}\left(b\right)=\{\begin{array}{cc} -\infty & b\leq 0\\ \left\lfloor {\text{log}}_{10}\left(b\right)\right\rfloor & \text{otherwise} \end{array}$$

Note that we do use the *floor* function instead of the *ceiling* one proposed in [10]. This operation do not change the intended usage in the original algorithm, while permitting to easily separate those bitflips that constantly changes (*e*.*g*., b[i]=1 implies that for each frame the *i*-th bit is different from the previous one). However, it is still unclear whether a more accurate function to replace the magnitude may provide a better base for the variable-splitting heuristic.

**Figure:**
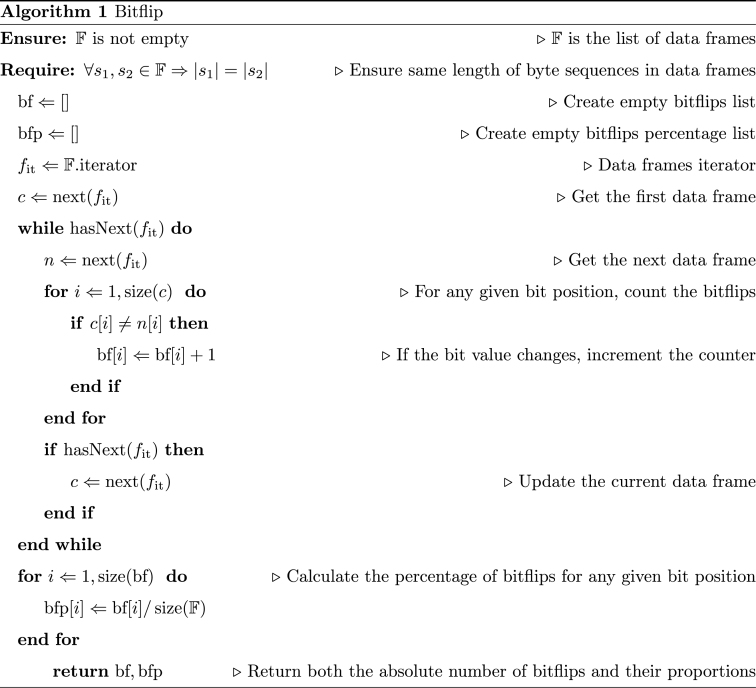

**Fig. 5:**
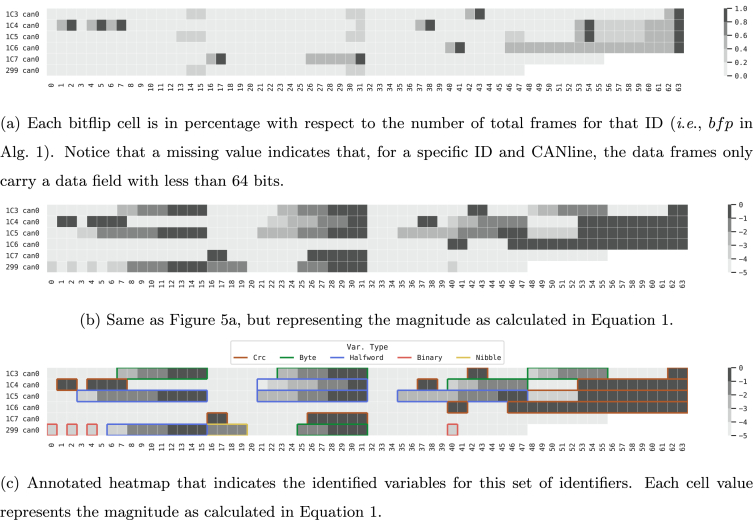
Sample bitflips heatmaps for vehicle Opel Corsa (See Table 2) with different level of information.

Fig. 5 reports a few sample heatmap for the bitflips of the vehicle Opel Corsa (See Table 2). In the figure, each row represents, in percentage, the number of bitflips for each one of the bits of the sequence (up to 64, depending on the identifier). For each identifier, the darker the cell, the higher the number of bitflips with respect to the number of frames received for that specific ID and CANline, *i*.*e*., indicated in Alg. 1 with the returned variable bfp. Empty cells indicate that the data frames received are smaller than 64 bits (See Fig. 3). For each experiment (as reported in Table 2), a graphical representation of their bitflips (like the sample presented in Fig. 5) is available in the repository.

Having calculated the bitflips and their magnitudes for each packet, Alg. 2 extracts the data blocks (*i*.*e*., potential variables) from the binary sequence (*s*) and their corresponding bitflips (*b*). Each data block obtained from the heuristic described in Alg. 2 may represent a variable.

**Figure:**
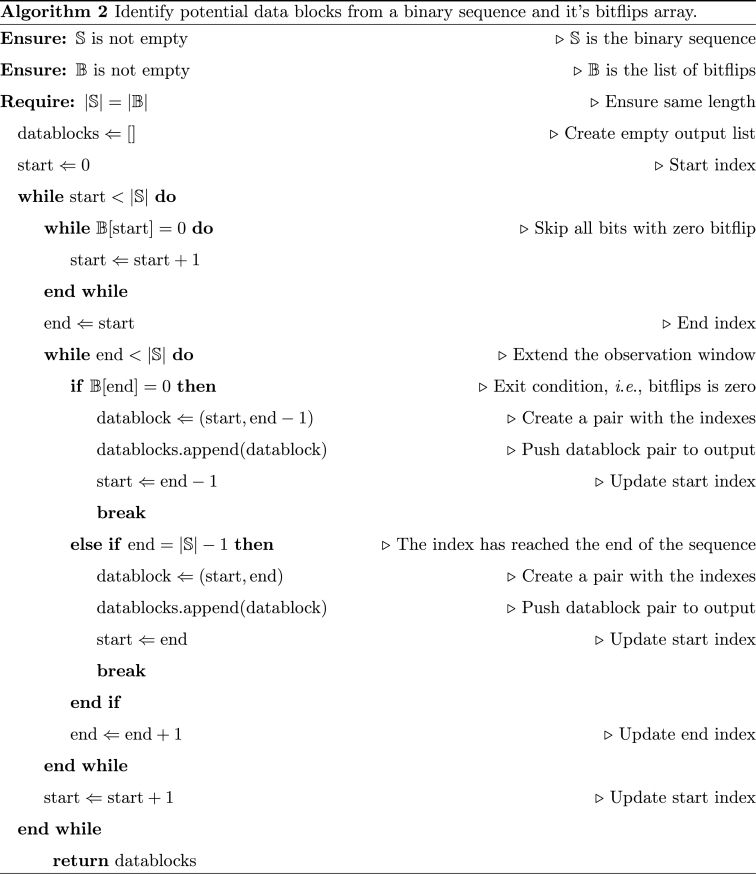

Nevertheless, as reported by [4,9,10], among others, car manufacturers tend to implement naïve protection strategies in the form of CRCs and counters. For example, a counter may be used to order the correct sequence of frames, while a CRC may be used to detect random transmission errors.

Finally, the third phase of the process consists of taking the calculated data blocks and attempt to decode them according to their type. In the specific case of both CRCs and counters, their respective data blocks are ignored. At this stage, each block falls in one of the following categories. Let *s* and *e* be the start index and the end index of the block, respectively; S be the binary sequence, B the corresponding bitflips vector and M their magnitudes.

• If s=e the block is considered as binary.
• If there is a region (a,b)⇒s≤a<b≤e in S such that ∀i⇒2⋅B[i]≈B[i+1], the region (a,b) is considered a counter. The remaining parts, if any, are re-analyzed as separated blocks.
• If the average bitflip value is between 0.5 and ± its standard deviation, the block is considered a crc.
• If the length of the block is between 1 and 4 (1<e−s≤4) the block is considered a nibble.
• If the length of the block is between 4 and 8 (4<e−s≤8) the block is considered a byte.
• If the length of the block is between 8 and 16 (8<e−s≤16) the block is considered a halfword.
• Otherwise the block is considered a word.

The division between nibble, byte, halfword, and word is instrumental to the analysis. To improve the comparability of the variables, they have been grouped according to their size (i.e., their values ranges).

### 2.5 Data characterization

This section focuses on providing sample insights on the proposed datasets. Each figure, table, or measure proposed in this section is available in the source code repository.

**Fig. 6:**
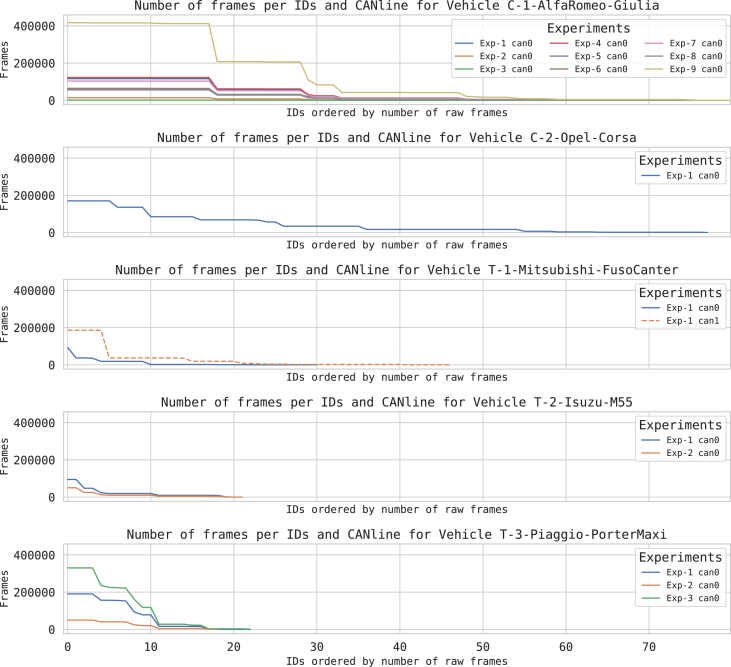
Number of data frames intercepted for each vehicle, CANline and experiment (Table 2), the series have been sorted and includes only those IDs for which there is at least one data variable obtained from the data extraction algorithm, as specified in Section 2.4.

**Fig. 7:**
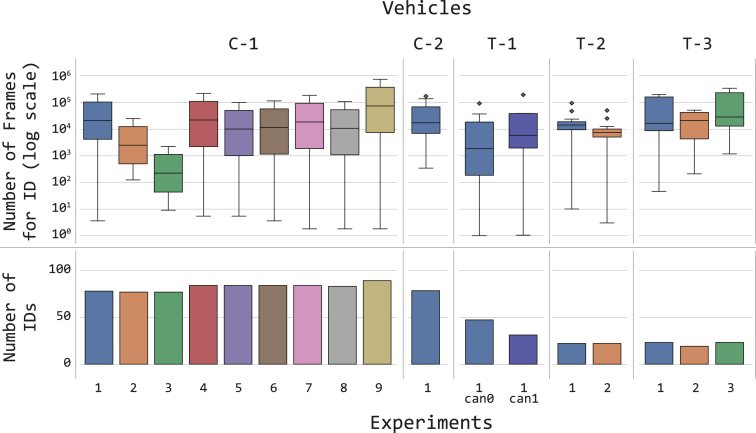
Statistical information regarding the number of ECU identifiers for each vehicle and experiment. The bottom axis presents the unique count of ECU identifiers, while the top axis reports the boxplots that describe the distributions of data frames for each vehicle, experiment and ECU identifier. Note that for vehicle T-1 there are two CAN lines.

The code repository, for each vehicle, contains both the source code and the generated statistics to verify these metrics. For example, Fig. 6 presents the distribution of the number of data frames per ECU ID, captured for each vehicle, experiment, and, where available, CANline. To be more precise, the vertical axes represent the number of data frames captured, while on the horizontal axis, the ECU identifiers that have been stripped of their names and sorted. In the figure, it is possible to notice that, independently from the vehicle, only a handful of IDs produces most of the data frames found in the network traffic. Fig. 7 presents these distributions as boxplots (on a logarithmic scale for visualization purposes).

**Fig. 8:**
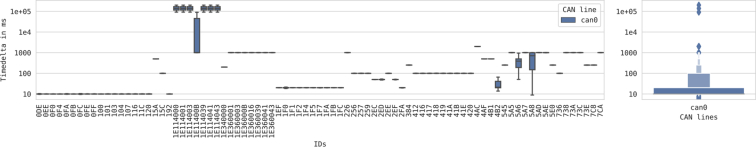
Interarrival frames times for vehicle Alfa Romeo Giulia (C-1). Values are in logarithmic scale.

The differences between the number of frames per ID can be explained by looking at the interarrival frames times per (ECU) identifier. Fig. 8 presents an example of such analysis for each ID of the Alfa Romeo Giulia (C-1, Exp-3).

### 2.6 Limitations

There are plain and noticeable limitations to the methods and algorithms used to decode the data. The following paragraphs will address the main one to provide clarifications.

First and foremost, OBD-II both refer to the physical connector (as specified by the SAE standard J1962 [3], formally the J1962 diagnostic connector) but also to the whole standard, also including the electric specifications and the communication protocol. In the context of this research, the data do not use the OBD-II protocol to request sensors data. However, the OBD-II connector has been used as a way to obtain direct access to the internal CAN networks.

#### 2.6.1 Hardware limitations

To the best of our knowledge, not all the vehicles offer public and unrestricted access to all the internal CAN networks through the OBD-II diagnostic connector, that is to say, apart from the aforementioned OBD-II querying protocol, the manufacturer is not required to provide direct access to the internal networks. For example, as cited before in Table 1, in at least one scenario was necessary to have direct physical access to the CAN network wires to be able to intercept the traffic.

#### 2.6.2 Sofware limitations

To the best of our knowledge, there are libraries and projects that offer the services of connecting, reading, and decoding vehicle data [11]. However, these libraries are based on the OBD-II querying protocol, which provides decoded data within a user-friendly interface. In contrast, the approach inheres proposed aims to bypass this protocol by providing data frames traces collected directly from the CAN networks through the OBD-II port. To be precise, the OBD-II only provides a subset of the information, while the CAN provides access to all the communications in the vehicle. However, older vehicles might not have access to the CAN network through this connector. Thus it will be required to identify, peel, and connect directly to the network in order to use it.

**Fig. 9:**
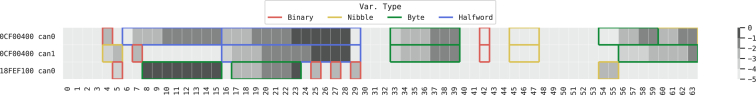
Bitflips magnitude heatmap for vehicle T-1, limited to the ECU identifier that carries the information regarding the (RPM) and speed sensors.

Since the exact match between the data encoding and the names and values of the sensors is usually proprietary, this research relies on the FMS datasheet, where and when available. Generally speaking, the FMSs are considered intellectual property and usually have a high market value. For example, in the case of the Mitsubishi commercial truck (T-1), where the FMS has been made available by the owning company, it was possible to validate the output obtained from the heuristic algorithm of Section 2.4 with the information provided in the FMS. In this case, it appears to be clear that there are noticeable differences regarding the identified and decoded variables and the actual sensors data specifications. Consider both Table 3 and Fig. 9 as base for this validation process. FMS specifications indicate that the speed, registered as a decimal number, is encoded with the ECU identifier 18FEF100 using bits 16–23 for the integer part and 8–15 for the decimal part. Despite having correctly located these two regions (see Fig. 9), the decode heuristic also identified 5 more variables, which might be used as support variables by the ECU. Correspondingly, the (RPM) data are encoded in the 32–39 (integer part) and 24–31 (decimal part) regions of 0CF00400 ECU identifier, while the algorithm also identified multiple locations that can be considered as variables.

To the best of our knowledge, unless indicated by the FMS datasheet, there are no heuristics that recognizes two components of the same variable, such as the integer and the fractional parts.

## References

[1] Zago M., Longari S., Tricarico A., Carminati M., Gil Pérez M., Martínez Pérez G., Zanero S. (2020). ReCAN Data - Reverse Engineering of Controller Area Networks.

[2] Zago M., Longari S., Tricarico A., Carminati M., Gil Pérez M., Martínez Pérez G., Zanero S. (2019). ReCAN Source - Reverse Engineering of Controller Area Networks. https://github.com/Cyberdefence-Lab-Murcia/ReCAN.

[3] SAE International Surface Vehicle Recommended Practice (Jul. 2016). SAE J1962: Diagnostic Connector.

[4] Le V.H., den Hartog J., Zannone N. (2018). Security and privacy for innovative automotive applications: a survey. Comput. Commun..

[5] International Organization for Standardization, Geneva (Apr. 2016). CH, ISO 15765-2: Road Vehicles – Diagnostic Communication over Controller Area Network (DoCAN) – Part 2: Transport Protocol and Network Layer Services.

[6] SAE International Surface Vehicle Recommended Practice (Aug. 2018). SAE J1939: Serial Control and Communications Heavy Duty Vehicle Network - Top Level Document.

[7] Evenchick E. (Jan 2017). CANtact. https://linklayer.github.io/cantact/.

[8] Linux Kernel (Feb 2018). Can Utils. https://github.com/linux-can/can-utils.

[9] Markovitz M., Wool A. (2017). Field classification, modeling and anomaly detection in unknown CAN bus networks. Vehicular Communications.

[10] Marchetti M., Stabili D. (2019). READ: reverse engineering of automotive data frames. IEEE Trans. Inf. Forensics Secur..

[11] Smith J.M. (2016). Awesome Vehicle Security. https://github.com/jaredthecoder/awesome-vehicle-security.

